# A high‐efficiency and stable perovskite solar cell fabricated in ambient air using a polyaniline passivation layer

**DOI:** 10.1038/s41598-021-04547-3

**Published:** 2022-01-13

**Authors:** Dong In Kim, Ji Won Lee, Rak Hyun Jeong, Jin-Hyo Boo

**Affiliations:** 1 Department of Chemistry, Sungkyunkwan University, Suwon, 440-746 Korea; 2 Institute of Basic Science, Sungkyunkwan University, Suwon, 440-746 Korea

**Keywords:** Chemical engineering, Surface assembly, Chemical engineering

## Abstract

Over the past number of years, the power conversion efficiency of perovskite solar cells has remained at 25.5%, reflecting a respectable result for the general incorporation of organometallic trihalide perovskite solar cells. However, perovskite solar cells still suffer from long-term stability issues. Perovskite decomposes upon exposure to moisture, thermal, and UV-A light. Studies related to this context have remained ongoing. Recently, research was mainly conducted on the stability of perovskite against non-radiative recombination. This study improved a critical instability in perovskite solar cells arising from non-radiative recombination and UV-A light using a passivation layer. The passivation layer comprised a polyaniline (PANI) polymer as an interfacial modifier inserted between the active layer and the electron transport layer. Accordingly, the UV-A light did not reach the active layer and confined the Pb^2+^ ions at PANI passivation layer. This study optimized the perovskite solar cells by controlling the concentration, thickness and drying conditions of the PANI passivation layer. As a result, the efficiency of the perovskite solar cell was achieved 15.1% and showed over 84% maintain in efficiency in the ambient air for one month using the 65 nm PANI passivation layer.

## Introduction

The field of solar cells, which is attracting attention as a renewable energy source, requires low-cost, high-efficiency, and stability characteristics. The performance of silicon solar cells is close to its theoretical limit and has high stability. Nevertheless, it is difficult to apply these cells to electronics, clothing, and construction products that have many curves and transparent characteristics. To overcome this, organic solar cells and dye-sensitized solar cells have been studied. However, limitation related to their efficiency, stability, and durability remain. Perovskite material has an ABX_3_ crystal structure. The A site cation is CH_3_NH_3_, the B site cation is Pb^2+^, and the x site anion is I^−^ in CH_3_NH_3_PbI_3_ (MAPbI_3_). MAPbI_3_ has attracted attention as a light-absorbing resource for next-generation solar cells^1–5^ due to its high absorption coefficient^6^, tunable bandgap^7^, long carrier diffusion length^8–11^, and low-cost^12,13^. Studies on sequential deposition^14,15^, solution processes^16–18^, vapor deposition^19^, and vapor-assisted solution processes^20^ have been developed to obtain high-quality MAPbI_3_. However, unbalanced carrier-mobility switches the direction and speed of the voltage sweep and leads to current density–voltage (J–V) hysteresis in mesoscopic structures^21,22^. To overcome this problem, charge transport material and charge transport layer/MAPbI_3_ interface studies^23–26^ and perovskite material studies^27,28^ have been conducted . In addition to forming a planar structure that eliminates the mesoporous (MP) layer which causes the J–V hysteresis problem, an inverted structure that changes the direction for reaching the electrodes of electrons and holes were studied, in which the efficiency of the perovskite solar cell (PSC) realized values higher than 23%^29–34^. To date, experiments have most often been conducted in laboratory conditions in the absence of moisture and oxygen such as inert atmosphere. These processes and systems are expensive to perform because they relate to commercialization conditions that demand high stability with the additive-free hole transporter materials^35,36^, durability, and excellent performance, as well as large-scale, low cost, and ambient air manufacturing. To meet the above conditions, large-scale deposition techniques^37–40^ and an encapsulation process^41–44^ were studied. Research regarding the fabricating of PSCs in ambient air conditions is currently at the foundational stage. One important aspect of ambient air conditions processes is the formation of high-quality films of oxygen-sensitive MAPbI_3_ materials^45^. Recent studies considered the positive and negative aspects of the effect of moisture on MAPbI_3_ using different approaches. For example, a small amount of water was added to the MAPbI_3_ precursor solvent during the deposition process to create a compact film^46^. It is important to note that the MAPbI_3_ layer had been protected from moisture to help transfer the carrier using an interlayer at the interface between the MAPbI_3_ and the transport layers.

Non-radiative recombination caused through defect formation at the transport layer/MAPbI_3_ interface, has been identified as a cause of efficiency loss in the PSCs^47^. The causes of MAPbI_3_ defects include temperature, bias voltage, ultraviolet-A rays (UV-A), and moisture. The exact cause of these defects must be identified according to research conducted to date; accordingly, the defect site was shown as having been generated by metal (lead(II), Pb^2+^) and/or halide (I^−^) ions remaining at the transport layer/MAPbI_3_ layer interface^48^. Non-radiative recombination through a defect formed at the transport layer/MAPbI_3_ layer interface can reduce optical properties and cell parameters, and can lead to J–V hysteresis and the long-term stability of J–V characteristics^49^. To maintain cell performance and stability, it is essential to reduce, the loss of non-radiative recombination at the transport layer/MAPbI_3_ layer interface. Recent studies have reported that non-radiative recombination in the device using the passivation layer at the interface was able to improve device stability^50^. Pb^2+^, which remains as a junction at the transport layer/MAPbI_3_ interface, is being studied to reduce defects using electron donor materials or Lewis-base materials. Representative materials such as polymers PVP^51^ and PCDTBT^52^ polymers, and devices have been found to increase carrier lifetime and voltage when passivated with Lewis basic thiophene and pyridine. Other organic passivation materials with fullerenes include PCBM^53^, graphene^54^ and PMMA^55^. Beside the chemical passivation, a physical passivation can also improve the stability of PSCs^56,57^.

MAPbI_3_ materials comprise organic and inorganic hybrids. MP-TiO_2_ PSCs have decreased stability under UV-A light conditions due to the changes in MP-TiO_2_ electron transport layers (ETL) widely used in high-performance PSCs^58^. When the MP-TiO_2_ layer is exposed to UV-A light, the photogenerated holes react with the oxygen that is absorbed in the surface oxygen vacancies this causes to deeply re-collect and gives rise to charge recombination. In the present study, an ultra-thin polyaniline passivation layer (PPL) was inserted into the ETL/MAPbI_3_ interface to manufacture a passivated PSC. The PPL trapped Pb^2+^ ions and absorbed light in the UV-A region improved the performance of the PSC, and reduced the interface carrier recombination. In addition, the P-type doping effect of an N-type ETL using a polyaniline (PANI) material caused a voltage increase through the enhancement of the overall Fermi-energy level in the device. A passivated PSC with 15.1% efficiency and an outstanding Voc of approximately 0.99 V was achieved. This study concludes that PPL can suppress the charge state of a Pb^2+^ defect at the ETL/MAPbI_3_ interface, thereby effectively reducing non-radiative recombination.

## Results and discussion

PANI polymers are classified into leucoemeraldine (fully reduced state), emeraldine (intermediate state), and nigraniline (fully oxidized state) according to the degree of oxidation and reduction. In the process of preparing PANI solution, PANI of leucoemeraldine state seems to have been oxidized and changed to emeraldine state. Therefore, PPL can absorb light from UV-A and visible light in a specific region. This was because MAPbI_3_ was decomposed by light in the UV-A region. Emeraldine PANI absorbed light in the UV-A region, which protected the MAPbI_3_ from being decomposed by UV-A light. PANI is classified as an intractable polymer, particularly when in the form of a conductive polymer. There are three ways in which to heighten the molecular weight of a PANI solution state (1) acid, (2) blend-solvent, and (3) an organic solvent. In this study, the ETL comprised TiO_2_, which was dissolved by an acid via the reaction shown in Eq. (1).

1 $$ {\text{TiO}}_{2} + \, 4{\text{HCl }} \to {\text{ TiCl}}_{4} + \, 2{\text{H}}_{2} {\text{O}} $$

**Figure 1:**
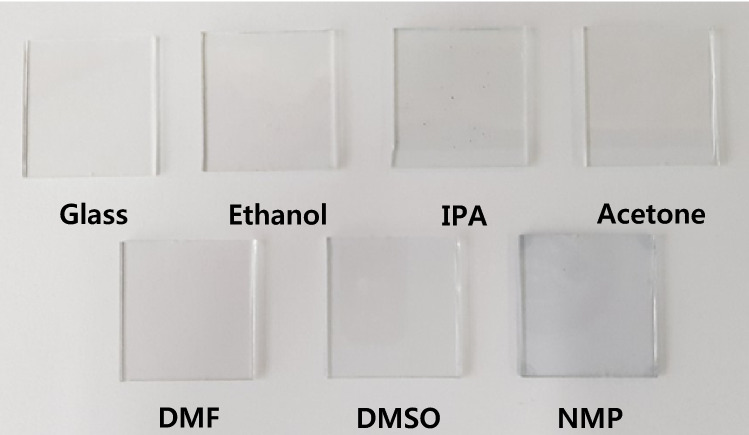
Graphical images of the PANI passivation layer (PPL), based on the solvent used.

An acid could not, however, be used as a solvent, accordingly, this study focused on organic solvents. In existing research, it was shown to dissolve well in polar solvents with strong hydrogen-bonding groups, such as cresols, phenols, trifluoroethanol, DMSO, and DMF. However, contrary to expectations, it still did not dissolve. The present study attempted to dissolve in ethanol and 2-propanol, but the same outcome was obtained. PANI powder appeared to have been dissolved in DMSO and DMF, however, it gradually begins to precipitate over time. Conversely, in the NMP solvent, the phenomenon mentioned above was not observed. A 1% organic solvent was able to dissolve PANI. Figure 1 shows the graphical images of PANI powder dissolved in various organic solvents before being coated onto a glass substrate. When PANI dissolved in NMP was coated onto a glass substrate, the entire glass substrate became a light-blue color. Contrastingly, no significant difference was observed on the glass substrate when using other solvents. The transmittance was measured to confirm the advantages of the PPL while maintaining the high transparency imbued by the glass electrode.

**Figure 2:**
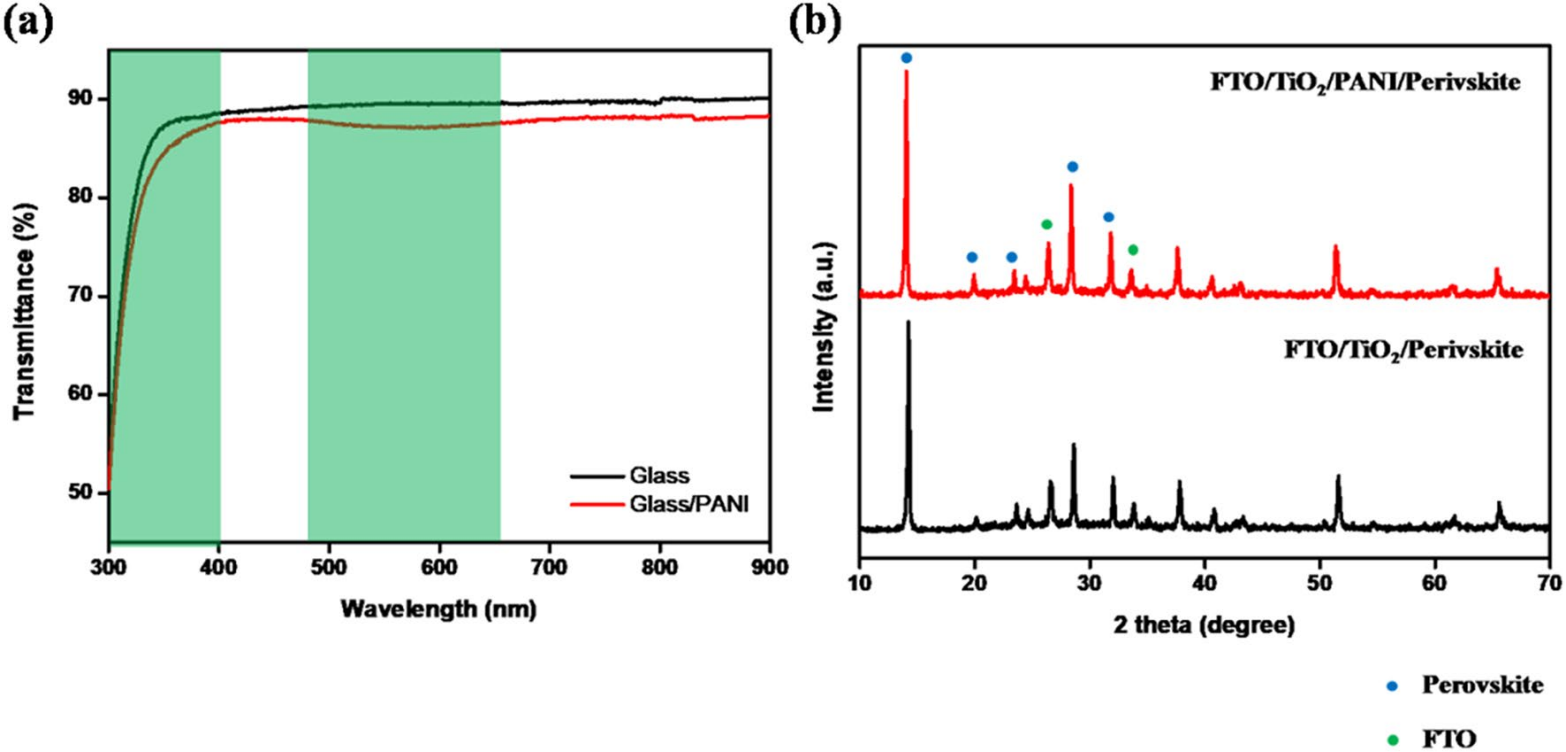
(**a**) The UV–Vis transmittance of glass with the PPL. (**b**) X-ray diffraction of the perovskite layer with/without PPL.

Figure 2a shows the transmittance of the PANI/glass substrate. Unlike that of the glass substrate, the transmittance curve of the PANI/glass substrate has changed. In the case of a glass substrate, the transmittance was slightly below 90% in the visible light region, when the PPL was deposited onto the glass substrate, the transmittance was reduced by approximately 1–2%. The PANI/glass sample showed lower transmittance in the UV-A range compared with the glass substrate. The PPL absorbed light within the UV-A region, therefore the transmittance of the PANI/glass sample in the range of 300–350 nm was significantly lower than for the glass sample. However, PPL absorbed in the visible region as well, which is the absorption region of perovskite. The PPL absorbed light within the UV-A region, therefore, the transmittance of the PANI/glass sample in the range of 300–350 nm was significantly lower than for the glass sample. The current density (Jsc) of the PSC may have decreased when the PPL was inserted into the ETL/MAPbI_3_ interfaces. However, the decrease in light amount of UV-A region improved the stability of the PSC. The XRD measurements for the phase identification of the MAPbI_3_ films on the CP-TiO_2_/FTO substrate, with and without PANI, are shown in Fig. 2b. The diffraction peaks of PbI_2_ that appeared at 12.6°, 34.3°, 39.5°, and 52.3° were assigned to the (001), (012), (003), and (004) lattice planes. The diffraction patterns at 14.2°, 20.0°, 23.6°, 24.6°, 28.5°, 31.9°, 35.0°, 40.6°, and 43.2° were assigned to the (110), (200), (211), (202), (220), (310), (312), (224), and (314) lattice planes of the MAPbI_3_. The MAPbI_3_ crystals were formed via the reaction shown in Eq. (2).

2 $$ {\text{PbI}}_{2} + {\text{ CH}}_{3} {\text{NH}}_{3} {\text{I }} \to {\text{ CH}}_{3} {\text{NH}}_{3} {\text{PbI}}_{3} $$

If the amount of MAPbI_3_ precursor was not sufficient for enabling a reaction, the PPL would adversely affects the formation of MAPbI_3_, and unreacted PbI_2_ would remain in the MAPbI_3_ film, this would present as an impurity in the XRD results. The MAPbI_3_ layer was deposited onto the CP-TiO_2_/glass sample and almost PbI_2_ impurities were not identified from the XRD results. The PANI/CP-TiO_2_/glass sample indicated the same results. This meant that the PPL did not affect the reaction in which the MAPbI_3_ crystallized.

**Figure 3:**
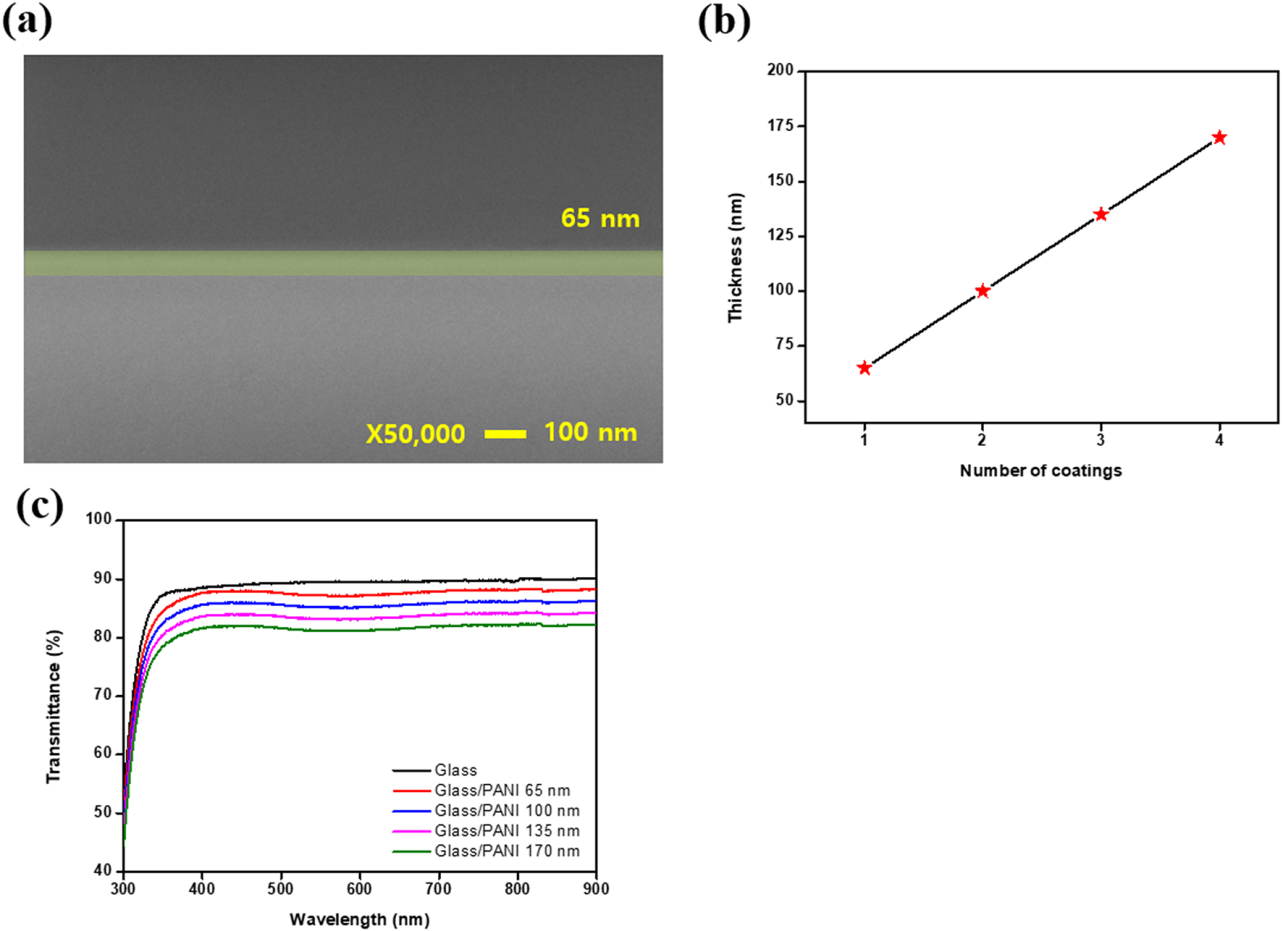
(**a**) The SEM image of the PPL (cross section image). (**b**) The thickness of the PPL and (**c**) the transmittance of the PPL according to its thickness.

Figure 3a shows the SEM cross-section image of the PPL. The thickness of the PPL is 65 nm and changes according to the number of coatings, as is summarized in Fig. 3b. As the number of coatings increased, the thickness of the PPL increased by 35 nm. After the number of coating was four times, a thickness of 170 nm was obtained. If the PPL is too thick, the transmittance will be lowered and the advantages of the glass electrode will be undermined. Figure 3c shows the transmittance according to the thickness of the PPL. As the number of coatings increased, the transmittance gradually reduced by 2% as indicated by the formation of the blue color on the PANI material.

**Figure 4:**
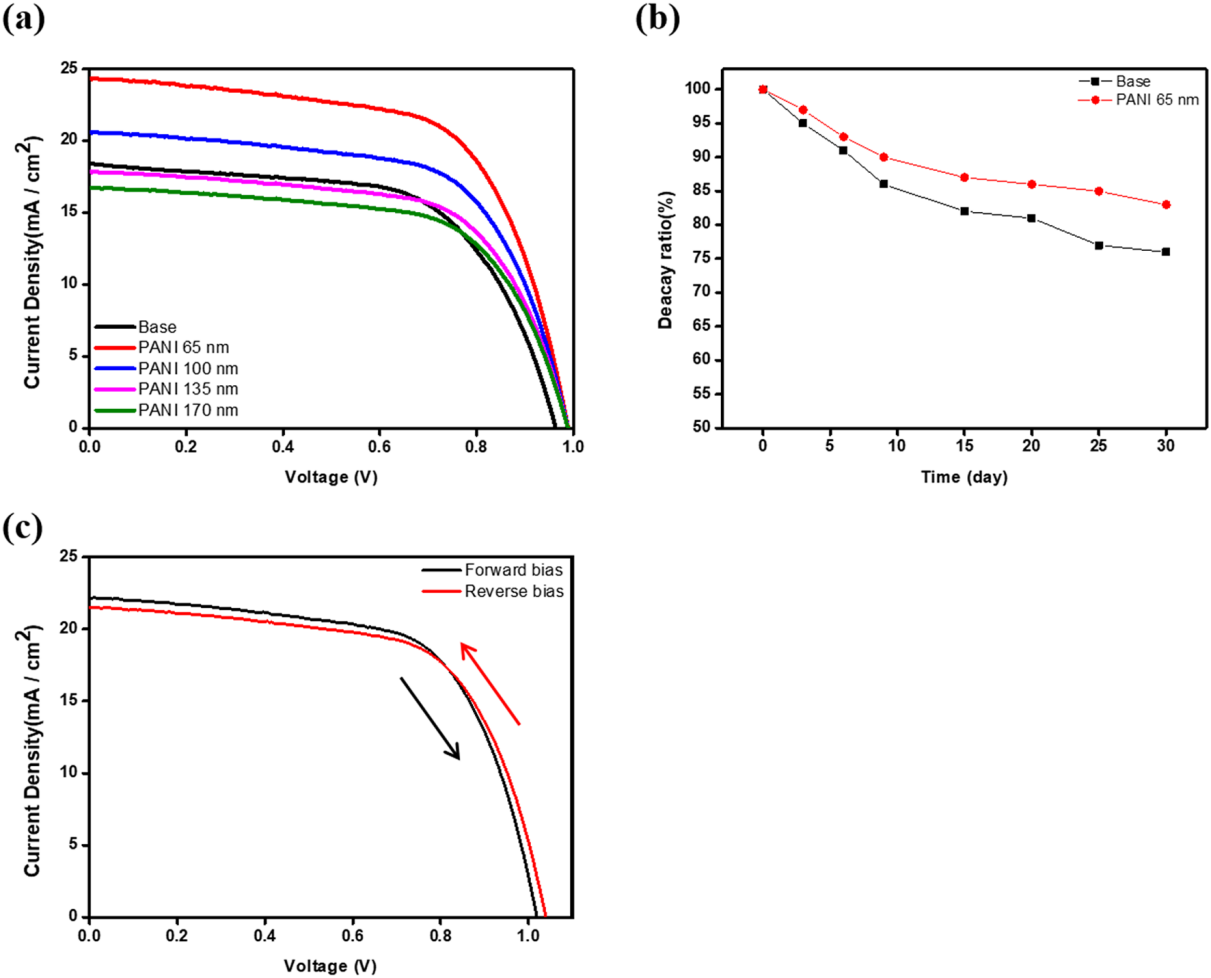
(**a**) The J–V curve and (**b**) the time-dependent evolution of efficiency (**c**) the J–V curve hysteresis of the perovskite solar cell with the PPL.

Figure 4a shows the details of the performance parameters of the PSCs as summarized in Table 1. One important effect of the PPL was that it increased the Voc. The voltage was improved due to the increase of the overall Fermi-energy level due to the P-type PPL doping effect of the N-type ETL. The Voc of the base sample was 0.96 V, which increased to 0.99 V with a 65 nm-PPL sample, a relative increase of 0.03 V. In addition, the Jsc of the 65 nm-PPL sample was increased to be 5.76 mA/cm^2^ compared with the base sample. Concurrently, the fill factor (FF) of the passivated PSC was minimally enhanced (by 2%), which this study ascribed to a decreased series resistance. Accordingly, a PSC with 65 nm-PPL sample was obtained at 15.1% of power conversion efficiency (PCE). When the PPL became thicker, the Jsc was slightly reduced due to a transmittance reduction in the visible region. As a result, the PCE gradually decreased the thickness of the PPL exceeds 65 nm-PPL. Figure 4b confirms the effect of the PPL on the non-radiative recombination of the base and 65 nm-PPL samples. Figure 4b shows the efficiency variation was measured for one month with the light blocked. The base sample lost 24% efficiency over the month, while the 65 nm-PPL sample’s efficiency was reduced by only 16%. The Pb^2+^ ions of the oxidation-decomposed MAPbI_3_ moved to the ETL/MAPbI_3_ interfaces, which affected carrier lifetime and mobility, and led to hysteresis. As a result, more Pb^2+^ ions were produced by oxidation over time, and the performance of the PSC decreased more rapidly. The PANI polymer material had a large number of lone pair electrons, which confined Pb^2+^ ions. The efficiency reduction amount of the 65 nm-PPL sample was smaller compared with the base sample. Figure 4c shows the minimal hysteresis of the 65 nm-PPL sample with respect to the voltage sweep.

**Table 1:** Photovoltaic performance parameters summarized J–V measurements with different PPL thickness.

Sample	Jsc (mA/cm^2^)	Voc (V)	FF (%)	PCE (%)
Base	17.95	0.96	62.1	10.7
65 nm	23.71	0.99	64.2	15.1
100 nm	20.10	0.98	64.1	12.7
135 nm	13.40	0.98	63.2	11.0
170 nm	16.30	0.98	64.4	10.3

**Figure 5:**
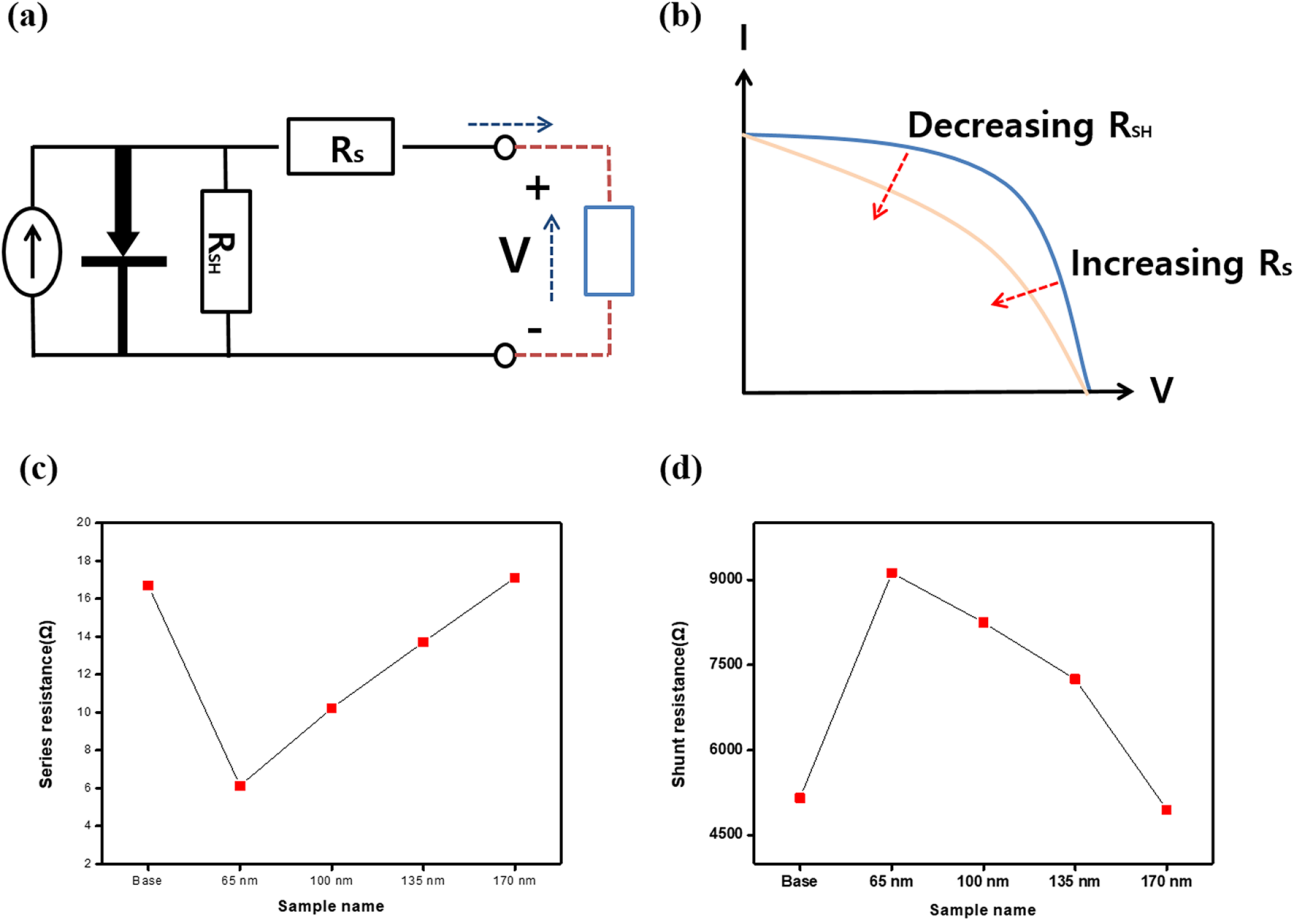
(**a**) The circuit and (**b**) the fill factor curve of the solar cell and (**c**) the series resistance and (**d**) the shunt resistance of the perovskite solar cell with PPL.

Figure 5a shows the solar cell circuit. To obtain a high-efficiency in the solar cell, the series resistance should be low and the shunt resistance should be high. If the series resistance is high, electrons cannot move smoothly along the circuit, and if the shunt resistance is low, leakage current will occurs, resulting in PSCs with poor efficiency and stability. These changes also affected the fill factor (FF) curve (see Fig. 5b). If the series resistance was high or the shunt resistance was low, the FF and the maximum output of the PSC would decrease. Based on the presence and the thickness of the PPL, the series and shunt resistances are shown in the Fig. 5c,d, and the values are summarized in Table 2. The series resistance value of the base sample was 16.70 Ω and the series resistance value decreased to 6.12 Ω when the 65 nm-PPL was inserted. As the PPL became thicker, the series resistance increased. The 170 nm-PANI sample obtained a value of 17.12 Ω, which was higher compared with the base sample. The shunt resistance value of the base sample was 5157.43 Ω and the was enhanced to 9122.47 Ω when the 65 nm-PPL was inserted. As the thickness of the PPL increased, the shunt resistance gradually decreased. The 170 nm PPL sample obtained a value of 4952.32 Ω, which was lower compared with the base sample. For the 65 nm-PPL sample, the ideal resistance value and the stability of the PSC were improved. The PL and impedance spectroscopy measurements were conducted to confirm the cause of cell parameter changes, based on the presence or absence of the PPL.

**Table 2:** Comparison of series and shunt resistances of perovskite solar cell with different PPL thickness.

	Base	65 nm	100 nm	135 nm	170 nm
R_s_ (Ω)	16.70	6.12	10.23	13.71	17.12
R_sh_ (Ω)	5157.43	9122.47	8247.47	7250.49	4952.32

**Figure 6:**
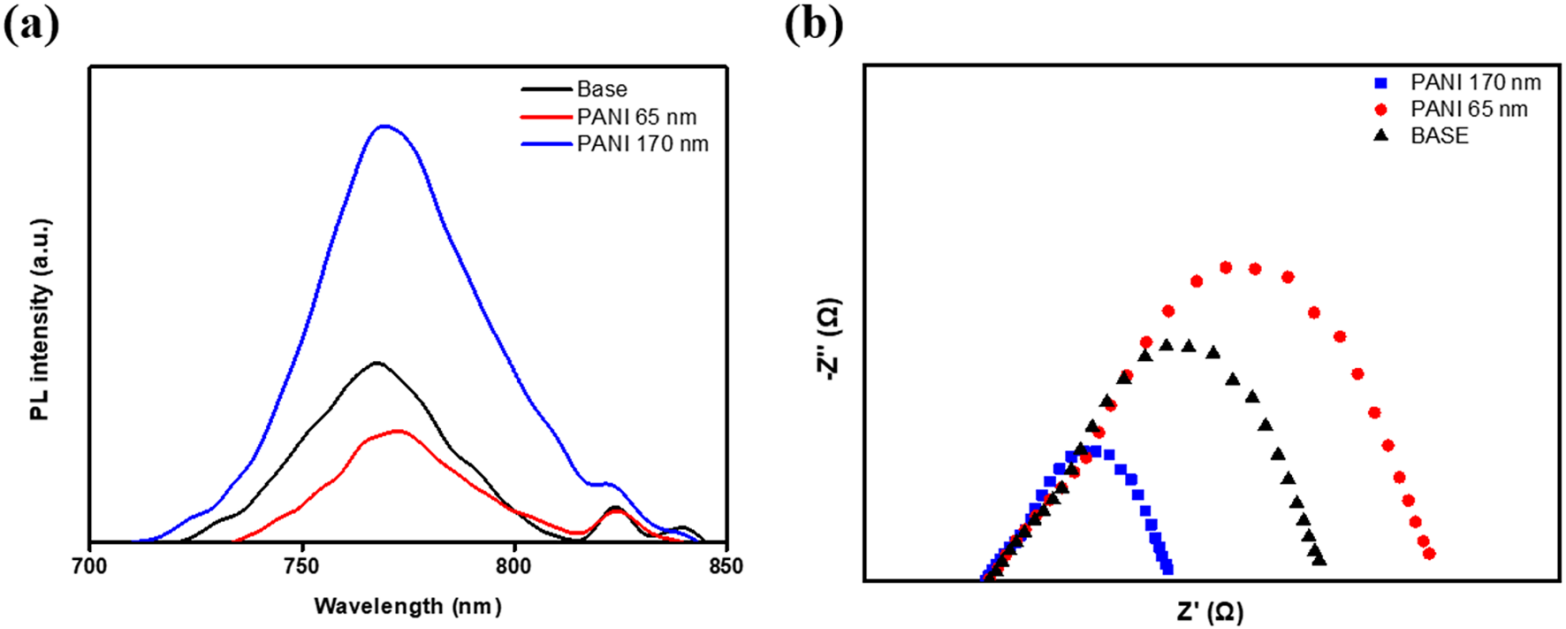
(**a**) The PL intensity and (**b**) the impedance of the perovskite solar cell with the PPL.

**Scheme 1:**
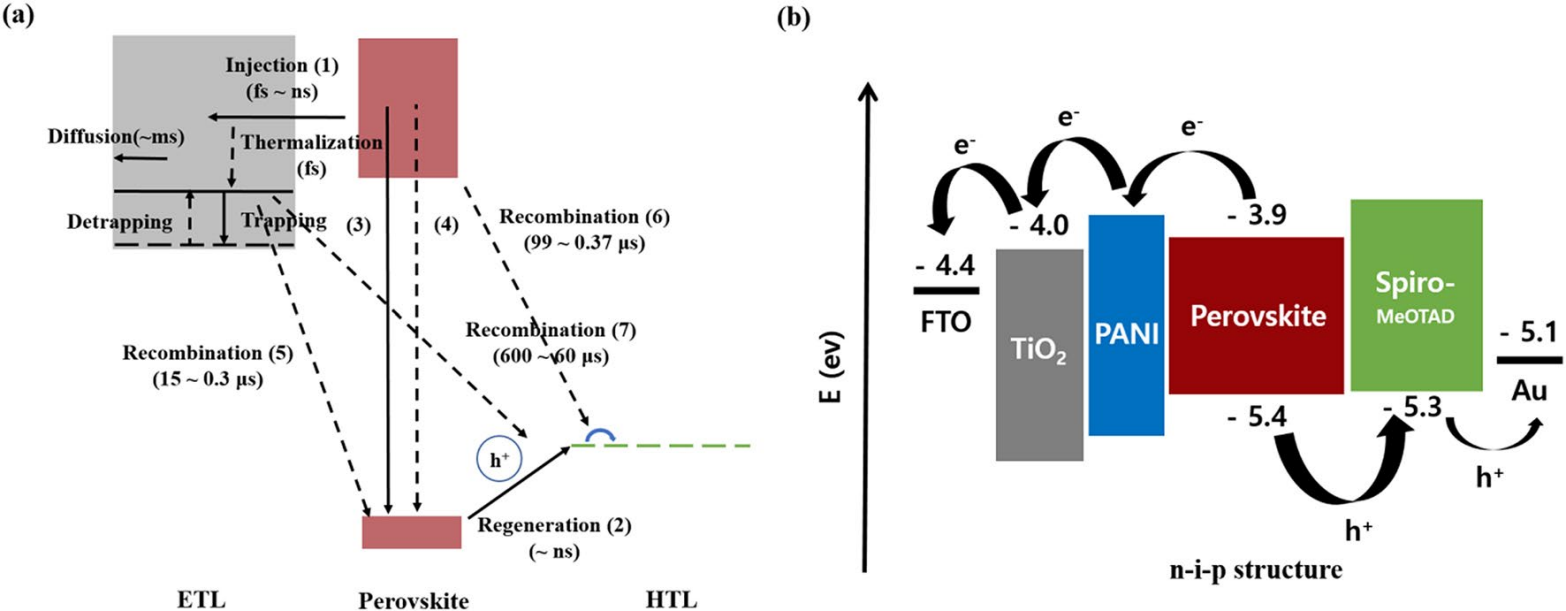
(**a**) Schematic diagram of carrier transfer processes in perovskite solar cells and (**b**) Energy band diagram of perovskite solar cells with PPL.

Figure 6a shows the PL curve. The area of the PL spectrum refers to the carrier recombination rate or carrier mobility. The results of the PL were affected by both the degree of exciton generation and the mobility of the carrier. For the 65 nm-PPL sample, the PL area was very small compared with the base sample. As the carrier lifetime increased, more electrons could reach the electrode, thereby increasing the current. Conversely, if the PPL was too thick, the recombination rate of the carrier was enhanced and the transmittance decreased. For this reason, the Jsc was reduced. The EIS measured the resistance at the interface (see Fig. 6b). The EIS included two resistors and capacitors (RC) arcs, one of which was related to the contact resistance of the interfaces at a high frequency and the other was attributed to the recombination resistance and chemical capacitance of the device at low a frequency. The EIS reflected the charge transfer resistance and recombination at the ETL/MAPbI_3_ interfaces and MAPbI_3_/counter electrode in the PSC field. The 65 nm-PPL sample exhibited smaller series resistance and larger recombination resistance compared with the base sample. This indicates that the carrier's transport ability had been improved and the recombination rate of the carrier had been reduced for the 65 nm-PPL sample. Conversely, the 170 nm-PPL sample exhibited larger series resistance and smaller recombination resistance compared to other sample. This result implies that the carrier's transport ability had been reduced and the recombination rate of carrier was increased for the 170 nm-PPL sample. This means that for the 170 nm-PPL sample, the carrier's transport ability had been reduced when the carrier recombination rate was increased. Scheme 1a summarizes the carrier transfer processes and Scheme 1b shows energy band diagram structure of the PSCs. The effect of the voltage enhancement via improvement of Fermi-energy level in the device (through the doping of a P-type material to an N-type ETL) was confirmed. If the thickness of the PANI layer is thin, separated electrons from the MAPbI_3_ are well injected into the(conduction band) ETL ((1): (e^−^ (electron)–h^+^ (hole)) MAPbI_3_ → e_cb(conduction band)_^−^ (ETL) + h^+^ (MAPbI_3_)) and the carrier recombination rate according to back charge transfer ((5): e_cb_^−^ (ETL) + h^+^ (MAPbI_3_) → recombination) is reduced due to the HOMO-energy level of the relatively high ETL. If the thickness of PANI layer becomes too thick, electrons cannot pass through the ETL and exciton annihilationn ((3): (e^−^– h^+^)perovskite → PL and (4): (e^−^–h^+^) MAPbI_3_ → non-radiative recombination) and back charge transfer ((6): h^+^ (HTL) + e^−^ (MAPbI_3_)) occurs at the PPL/MAPbI_3_ interface. The recombination resistance and carrier lifetime were improved through the insertion of a conducting PANI polymer, thereby improving the FF and Jsc. These effects resulted in improved PSC stability and efficiency. The Pb^2+^ ions, which represent an impurity generated by oxidation over time due to non-radiative recombination, was trapped in the PPL, thereby providing stability over time.

Table 3 summarizes the PSCs manufacturing in relation to atmospheric conditions and moisture. The research on manufacturing PSCs under atmospheric conditions in relation to oxygen is a very important in the context of commercialization, not only in terms of stability but also in relation to manufacturing costs. Existing research has attempted to add a hydrophilic material to a precursor solution or a coating process to resist oxygen effects. In addition, various studies have been conducted to improve the stability and efficiency while lowering the manufacturing costs of all manufacturing processes performed in the atmospheric conditions. The present research shows that all the manufacturing processes were conducted as part of the atmospheric process. Furthermore, it indicates the improvement of the stability and efficiency of the PPL, as well as its high level of performance when comparing the efficiency/cell area with other research teams.

**Table 3:** Efficiency comparison of the results of other group reported in ambient air process perovskite solar cell.

YYYY.MM	PCE (%)	Cell area (cm^2^)	Device structure	References
2015.04	10.1	0.24	ITO/PEDOT:PSS/Perovskite/PCBM/BCP/Ag	^59^
2015.09	18.0		ITO/PEDOT:PSS/Perovskite/PCBM/Ca/Al	^29^
2015.09	9.4	0.092	FTO/TiO_2_/Al_2_O_3_ + Perovskite/SWCNTs/PMMA/Ag	^60^
2016.10	12.0		ITO/PEDOT:PSS/Perovskite/PCBM/C_60_/BCP/Ag	^61^
2016.10	16.3	0.05	ITO/PEDOT:PSS/Perovskite/PCBM/PDINO/Al	^62^
2017.01	18.1	0.1	ITO/Poly-TPD/Perovskite/C_60_/BCP/Ag	^63^
2017.02	15.6	0.126	FTO/TiO_2_/Perovskite/ZrO_2_/Carbon	^64^
2018.01	18.7	4.85	ITO/PEDOT:PSS/Perovskite/C_60_/BCP/Ag	^65^
2018.12	17.1	0.1	ITO/PEDOT:PSS/Perovskite/C_60_/BCP/Ag	^66^
This work	15.1	0.16	FTO/TiO_2_/Perovskite/Spiro-MeOTAD/Ag	

## Conclusions

This study demonstrated a passivated MAPbI_3_ structure for suppressing defects at the interface of an ETL/MAPbI_3_ using a PPL. The PPL absorbed UV-A to prevent damage to the MAPbI_3_ layer and suppressed non-radiative recombination between the MAPbI_3_ and ETL interface. Increasing the stability and performance of the PSC represents a significant advantage, that is, the PPL's reduced carrier recombination rate. As a result, a Voc of 0.99 V was obtained, and a conversion efficiency of 15.1% was realized for the PSC. The PPL is generally applicable to other organic and organic–inorganic solar cells for improving their efficiency and stability. To improve PSC's stability, a variety of donor–acceptor materials can be used with PANI to suppress non-radiative recombination.

## Methods

### Preparation of materials

The synthesis of CH_3_NH_3_I powder: A methylamine (27.8 ml, 40 wt% in methanol, TCI) and hydroiodic acid (30 ml, 57 wt% in water, Sigma-Aldrich) mixture solution were stirred at 0 °C for 2 h. To remove the solvent of the synthesized CH_3_NH_3_I powder, the mixture was rotary evaporated at 50 °C for 1 h. The CH_3_NH_3_I powder was washed with diethyl ether and dried in an oven at 60 °C for 24 h.

Preparation of PANI solution: 0.1 mg of PANI (emeraldine base, Sigma-Aldrich) is dissolved in 1 ml of 1-Methyl-2-pyrrolidinone (NMP, 99.5%, Sigma-Aldrich), followed by stirring at 70 °C for 24 h.

### Fabrication of the PSCs

The fluorine doped tin oxide (FTO) substrate was etched with Zn dust and 2 M hydrochloric acid and cleaned with acetone and 2-propanol for 10 min respectively. The FTO substrate was dried with nitrogen and exposed to oxygen plasma treatment. To creat the TiO_2_ compact layer (CP-TiO_2_), 0.10 M titanium diisopropoxide bis (acetylacetonate) (Sigma-Aldrich, 75 wt%, in IPA, Signa-Aldrich) solution was deposited on the FTO substrate and annealed at 450 °C for 30 min. The PANI solution was then coated onto the CP-TiO_2_ layer. A mixture solution of 1.0 M PbI_2_ (1.0 M, Sigma-Aldrich) and 1.0 M CH_3_NH_3_I in *N,N*-Dimethylformamide (DMF), and dimethylsulfoxide (DMSO) at a ratio of 4:1 (v:v), was coated on the PPL and dried at 100 °C for 10 min. A Spiro-MeOTAD containing [28.8 μL of Spiro-MeOTAD (Lumtec) (72.3 mg/mL in chlorobenzene), 4-tert-butylpyridine (Sigma-Aldrich, 96% The TSFI stock solution (Sigma-Aldrich, 99.8%) was stirred for 24 h)] was coated on the MAPbI_3_ layer. A gold electrode was created using thermal evaporation equipment.

### Characterization

Film X-ray diffraction (XRD) (Bruker D8 Advance system) measurement was performed using Cu Kα radiation (λ = 1.5416 Å) with a 40 kV beam voltage and a 30 mA beam current. Scanning electron microscopy (SEM) was performed using a JEOL, JSM-7100F instrument. The transmittance and absorbance (ABS) characteristics were recorded using a UV–Vis-near-infrared (NIR) spectrometer (UV-3600, Shimadzu) in the wavelength range of 300–900 nm with an integrated sphere attachment. The photoluminescence (PL) was measured by using a FluroMate (FS-2) fluorescence Sperctrometer. The photovoltaic performance (SUN 2000) was achieved using a xenon lamp under an AM 1.5 filter at 100 mW/cm^2^ illuminations in open circuit conditions. The resistances of the PSCs were obtained using the Iviumsoft program. Electrochemical impedance spectroscopy (EIS) was performed in a frequency range from 1 MHz to 100 mHz using a SUN 2000 Instrument under an alternating current voltage with a perturbation amplitude of 10 mV was applied in the EIS measurements.
